# BRG1 Is Dispensable for Sertoli Cell Development and Functions in Mice

**DOI:** 10.3390/ijms21124358

**Published:** 2020-06-19

**Authors:** Shuai Wang, Pengxiang Wang, Dongli Liang, Yuan Wang

**Affiliations:** 1 Shanghai Key Laboratory of Regulatory Biology, Institute of Biomedical Sciences and School of Life Sciences, East China Normal University, Shanghai 200241, China; wangshuai2509@126.com (S.W.); wangpengxiang1900@163.com (P.W.); 2 Department of Animal Sciences, College of Agriculture and Natural Resources, Michigan State University, East Lansing, MI 48824, USA

**Keywords:** Sertoli cell, spermatogenesis, BRG1

## Abstract

Sertoli cells are somatic supporting cells in spermatogenic niche and play critical roles in germ cell development, but it is yet to be understood how epigenetic modifiers regulate Sertoli cell development and contribution to spermatogenesis. BRG1 (Brahma related gene 1) is a catalytic subunit of the mammalian SWI/SNF chromatin remodeling complex and participates in transcriptional regulation. The present study aimed to define the functions of BRG1 in mouse Sertoli cells during mouse spermatogenesis. We found that BRG1 protein was localized in the nuclei of both Sertoli cells and germ cells in seminiferous tubules. We further examined the requirement of BRG1 in Sertoli cell development using a *Brg1* conditional knockout mouse model and two *Amh-Cre* mouse strains to specifically delete *Brg1* gene from Sertoli cells. We found that the *Amh-Cre* mice from Jackson Laboratory had inefficient recombinase activities in Sertoli cells, while the other *Amh-Cre* strain from the European Mouse Mutant Archive achieved complete *Brg1* deletion in Sertoli cells. Nevertheless, the conditional knockout of *Brg1* from Sertoli cells by neither of *Amh-Cre* strains led to any detectable abnormalities in the development of either Sertoli cells or germ cells, suggesting that BRG1-SWI/SNF complex is dispensable to the functions of Sertoli cells in spermatogenesis.

## 1. Introduction

During mammalian spermatogenesis, haploid spermatozoa are continuously produced from spermatogonial stem cells (SSCs). This process requires a tightly regulated interplay between germ cells and various somatic supporting cells in the spermatogenic niche. Of these supporting cell types, Leydig cells, peritubular myoid cells, fibroblasts, and macrophages all locate at interstitium or immediately outside the seminiferous tubules. The exception is Sertoli cells, which stay within the tubules and interact directly with germ cells at various stages of spermatogenesis [1,2,3]. In mouse, Sertoli cells originally develop from SF1+ gonadal precursors at 11.5–12.5 days post coitum (dpc), marked with the expression of SOX9 and anti-Mullerian hormone (AMH) [4,5]. Immature Sertoli cells continue to proliferate until 12–17 days post-partum (dpp). Afterwards, Sertoli cells exit from cell cycle and enter a differentiation process which includes a cessation of proliferation, alterations in transcription and protein expression, and functional maturation [6,7].

During embryonic testis formation, Sertoli cells sequester male gonocytes inside of the newly formed seminiferous tubules and prevent them from entering meiosis [2]. In adult testis, Sertoli cells extend from basal lamina to the lumen of seminiferous tubules, and thus provide a structural scaffold for germ cells to move towards the lumen during differentiation [1,2]. In addition, the tight junctions between adjacent Sertoli cells and blood vessels constitute a blood-testis barrier (BTB), which prevents blood borne toxins from entering the tubules and thus offers germ cells with immune privilege [1,3]. Sertoli cells are also called “nurse” cells, as they produce a panel of factors and nutrients to support the successful progression of SSCs into spermatozoa [4,8,9]. Examples of secreted factors from Sertoli cells include retinoic acid (RA), bone morphogenic protein 4 (BMP4), stem cell factor (SCF), KIT ligand (KITL), and glial cell line-derived neurotrophic factor (GDNF), all of which are crucial to maintain the balance between SSC self-renewal and differentiation [2,10,11,12,13,14,15]. Sertoli cells are therefore vital to spermatogenesis, and their dysfunction may lead to failure of germ cell development [16,17]. Despite critical contribution from Sertoli cells to germ cell development, it is surprising that only about 60 Sertoli specific knockout mice had been generated. So far, it remains to be understood how the transcription of those secreted factors is regulated in Sertoli cells during germ cell development.

The SWI/SNF family of chromatin remodeling complex contains either BRM or BRG1 as the catalytic subunit to utilize energy from ATP hydrolysis to alter the chromatin architecture of target promoters, and thus plays a regulatory role in gene transcription [18]. For example, BRG1 modulates the expression of a number of genes that participate in cell proliferation, motility, and adhesion [19]. Interestingly, BRG1 bound to regulatory elements of *Wt1* and *Sox9*, two genes highly expressed in Sertoli cells, to regulate their transcription during development [20,21]. It, however, remains undefined whether BRG1 is required for Sertoli cell development and functions. BRG1 also plays important roles in cell proliferation and development. The reintroduction of BRG1 into ALAB breast tumor cell line, which carrying a defined mutation in the BRG1, induced growth arrest, suggesting an inhibitory role of BRG1 in cell growth [19]. *Brg1* knockout (KO) leads to early embryonic lethality prior to implantation, while a reduced level of BRG1 protein causes exencephaly due to abnormal cell proliferation [22]. In addition, *Brg1* heterozygote knockout mice are susceptible to mammary tumors which exhibit genomic instability [23]. Further, germline-specific ablation of *Brg1* results in defects in spermatogenesis and male infertility, due to disrupted DNA repair and abnormal chromatin modifications [24,25]. Compared to BRG1, altered cell proliferation is observed in the absence of BRM. However, *Brm* knockout males develop normally and are fertile, suggesting a functional difference of BRG1 from BRM containing SWI/SNF comp [26].

The number and functions of Sertoli cells in the adult testis determine both testis size and daily sperm production [6]. Given the critical roles of BRG1 in regulating the transcription of Sertoli cell-related genes and cell proliferation [20,21,22], we aim to understand whether the BRG1-SWI/SNF complex regulates Sertoli cell development and functions. We conditionally deleted the *Brg1* gene from Sertoli cells, using two different *Amh-Cre* strains. Compared to *Amh-Cre* mouse model from the Jackson Laboratory, *Amh-Cre* mice from European Mouse Mutant Archive (EMMA) displayed more robust Cre recombinase activities. However, no detectable abnormalities of spermatogenesis and fertility were observed in mice with complete *Brg1* deletion from Sertoli cells, indicating that *Brg1* gene is not required for Sertoli cell functions during spermatogenesis.

## 2. Results

### 2.1. BRG1 Is Expressed in Both Sertoli Cells and Germ Cells

**Figure 1:**
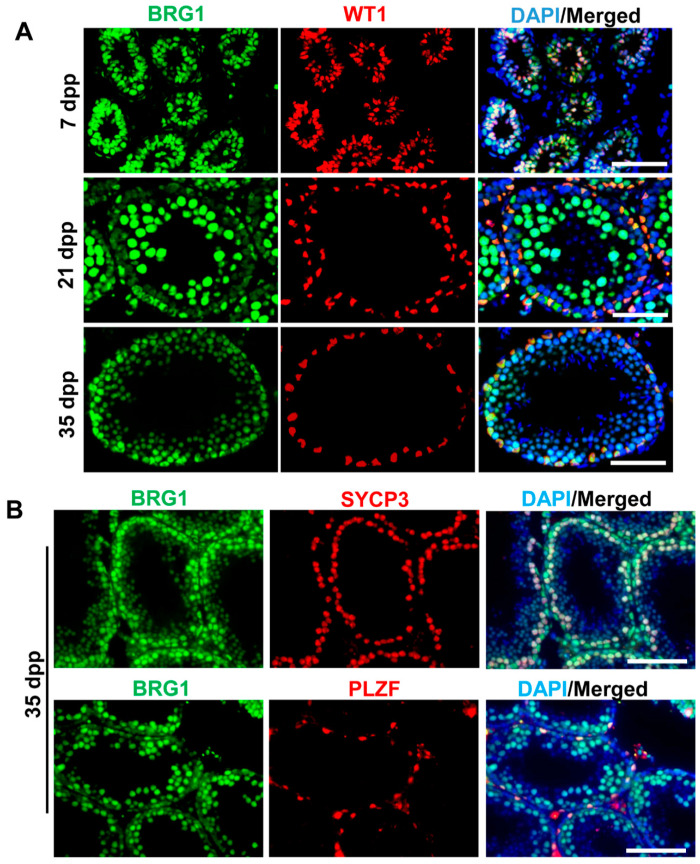
BRG1 protein is highly expressed in both Sertoli cells and germ cells. (**A**) Co-localization of BRG1 and WT1 was examined by IHF in testes from mice at various ages. Scale bars: 100 μm. (**B**) Co-localization of BRG1 and germ cell specific proteins PLZF and SYCP3 was examined by IHF in testes from mice at 35 dpp. Scale bars: 100 μm.

To investigate the role of the BRG1-SWI/SNF complex in Sertoli cells, we first examined BRG1 expression in testes during spermatogenesis with immunohistofluorescence (IHF) analyses. We observed that BRG1 protein was expressed at 7, 21, and 35 dpp in Sertoli cells, as displayed by its co-localization with WT1, a Sertoli cell specific marker (Figure 1A), suggesting a potential role of BRG1 in Sertoli development. In addition, consistent with published data [24,25], BRG1 was also detected in germ cells. The IHF staining of testicular sections from 35 dpp mice revealed that BRG1 was highly expressed in undifferentiated spermatogonia (BRG1^+^PLZF^+^ cells) and spermatocytes (BRG1^+^SYCP3^+^ cells). BRG1 was also detected in round spermatids (BRG1^+^SYCP3^-^ cells in the adluminal compartment of the seminiferous tubules), albeit at a lower level than that in spermatocytes. No BRG1 protein was found in elongated spermatids (BRG1^-^SYCP3^-^DAPI^+^ cells in the adluminal compartment of the seminiferous tubules) (Figure 1B).

### 2.2. Partial Brg1 Deletion in Sertoli Cells Does Not Affect Sertoli Cell Development

**Figure 2:**
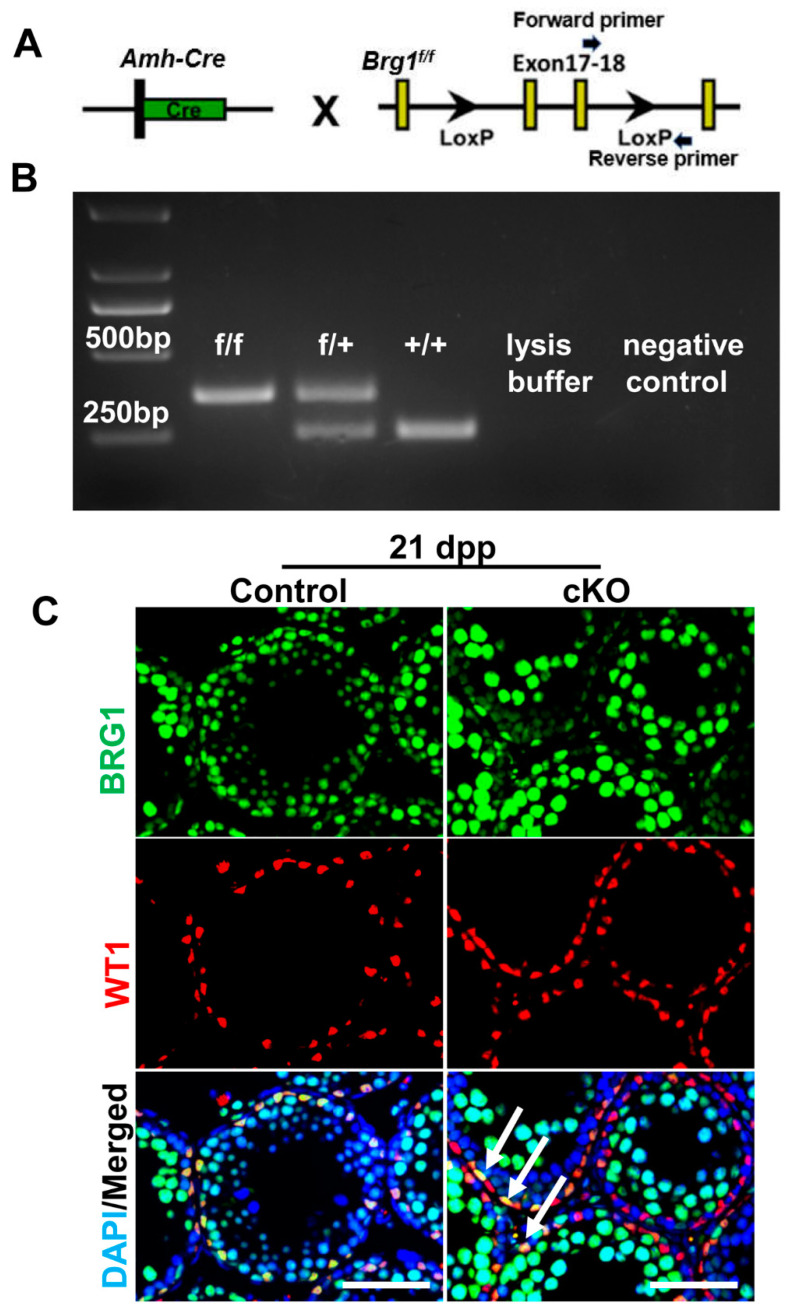
Partial deletion of *Brg1* in Sertoli cells by *Amh-Cre* mice from Jackson Lab. (**A**) Strategy to delete *Brg1* in Sertoli cells by *Amh-Cre* (Jackson) mice. Arrows indicate position of forward and reverse primers for genotyping analyses to detect *Brg1* floxed alleles. (**B**) An example of genotyping on offspring from *Brg1^f/+^* mice crossed with *Brg1^f/+^; Amh-Cre.* PCR on lysed mouse tail snips amplified the *Brg1* genomic locus to generate a 380 bp DNA band from the mutated region, containing a loxP site or a 250 bp band from wildtype locus. +/+: wildtype; f/+: heterozygous; f/f: homozygous. (**C**) BRG1 and WT1 proteins were examined by IHF in testicle sections from *Brg1* cKO mice (*Brg1^f/f^; Amh-Cre*) and their littermate controls at 21 dpp. Arrows point to WT1+ Sertoli cells with BRG1 expression. Scale bars, 100 μm.

To bypass the embryonic lethality caused by *Brg1* knockout, we next generated mice in which the *Brg1* gene was specifically disrupted in testicular Sertoli cells. Conditional *Brg1* knockout mice (*Brg1^f/f^*) were crossed with an *Amh-Cre* line from the Jackson Laboratory (Figure 2A,B). Cre expression in this *Amh-Cre* (Jackson) mouse strain starts around 14.5 dpc in Sertoli cells (see the Reference [27] in Materials and Methods). *Brg1* deletion efficiency in Sertoli cells was analyzed by examining BRG1 protein expression with IHF at 21 dpp. WT1 was used as a marker to label Sertoli cells. We found that BRG1 protein co-expressed with WT1 in a fraction of Sertoli cells (Figure 2C), suggesting that *Brg1* was only partially deleted in Sertoli cells from *Brg1^f/f^; Amh-Cre* (abbreviated as cKO: conditional knockout) mice. In addition, the numbers of WT1+ Sertoli cells in partial *Brg1* cKO mice were comparable to those in littermate controls (Figure 2C).

We thus decided to evaluate *Cre* recombinase activity in this *Amh-Cre* (Jackson) line. We crossed *Amh-Cre* (Jackson) mice with a commonly used *Rosa26-EYFP* reporter line, in which the EYFP is expressed upon CRE-mediated excision of a strong transcriptional termination sequence (triple SV40 polyadenylation sequence), flanked with two loxP sites before its coding sequence (see the Reference [28] in Materials and Methods). We found that *Amh-Cre* (Jackson) indeed activated *Rosa26-EYFP* expression only in testis, but not in other organs such as heart, brain, and kidney from *Rosa26-EYFP; Amh-Cre* mice (Figure S1A). However, IHF showed that EYFP protein was not detected in many WT1^+^ Sertoli cells (Figure S1B), thus confirming that this *Amh-Cre* (Jackson) line has inefficient CRE activity. This likely leads to the incomplete deletion of the termination sequence before *EYFP*.

### 2.3. Partial Deletion of Brg1 in Sertoli Cells Does Not Affect Spermatogenesis

The partial deletion of the *Brg1* gene could offer an opportunity to understand the functional mechanism of BRG1-SWI/SNF complex in Sertoli cell development. We thus continue to investigate the requirement for BRG1 in Sertoli cells during spermatogenesis using this partial *Brg1* deletion mouse model with the *Amh-Cre* (Jackson) line.

**Figure 3:**
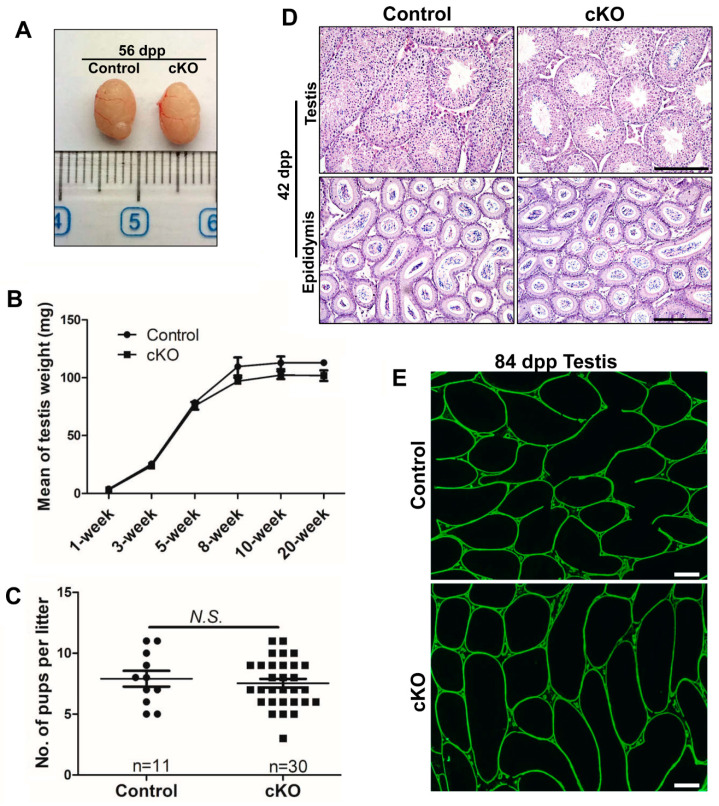
Partial deletion of *Brg1* does not affect Sertoli cell development and spermatogenesis. (**A**) Representative images of testes from control littermates and cKO mice at 56 dpp. (**B**) Mean weight of testes from control and cKO mice at different ages, calculated from 3–10 animals per group. Data represent mean ± SEM. Statistical analyses were performed using Student’s t-test. (**C**) The number of pups per litter, calculated from 11 litters from 4 control mice and 30 litters from 5 cKO males. Data represent mean ± SEM. Statistical analyses were performed using Student’s t-test. *N.S*.: no significance. (**D**) Histology study of testes and epididymides from control and cKO mice at 42 dpp. Scale bars: 200 μm. (**E**) BTB permeability was assayed with the biotin tracer (green) in testes from 84 dpp mice. Scale bars, 200 μm.

Testes from control and cKO littermates at different ages were analyzed. We did not find any significant differences in testis morphology, weight and body weight between the two groups at various stages (Figure 3A,B; Figure S2A). Neither did we detect any obvious change in numbers of pups per litter produced from breeding pairs with *Brg1* conditionally deleted male mice, compared to those from their littermate controls (Figure 3C).

The first wave of spermatogenesis is completed in mice at 35 dpp. Therefore, we performed histology study on testes and epididymides from mice at 42 dpp. We found spermatogenic cells at all stages in the seminiferous tubules from both cKO and control testes (Figure 3D). The testis and epididymis structure appeared to be normal, with partial *Brg1* deletion in Sertoli cells (Figure 3D). Similar numbers of sperm in epididymides from cKO and control mice were detected (Figure 3D). No obvious difference was observed between cKO and control testes in germ cell development from mice at either 14 dpp or 28 dpp (Figure S2B). These data suggest that spermatogenesis is normal in mice with partial *Brg1* deletion in Sertoli cells.

As tight junctions of Sertoli cells are part of the BTB, we next examined BTB integrity upon *Brg1* partial deletion in Sertoli cells. In this assay, a biotin tracer was injected into the interstitium of testis, and IHF on testis sections was performed using streptavidin conjugated with a green fluorochrome (Alexa Fluor 488). The disruption of BTB will lead to permeability of biotin tracer into the lumen of seminiferous tubules, which can be detected by streptavidin and exhibited by green signals in IHF. We found that the biotin tracer was restricted to the interstitial space in both cKO and control mice (Figure 3E), indicating that the BTB remains intact upon *Brg1* partial deletion in Sertoli cells. Taken together, these results suggest that partial *Brg1* conditional knockout in Sertoli cells does not affect Sertoli cell development and functions, spermatogenesis, and male fertility.

### 2.4. Efficient Brg1 Deletion in Sertoli Cells Does Not Affect Spermatogenesis

**Figure 4:**
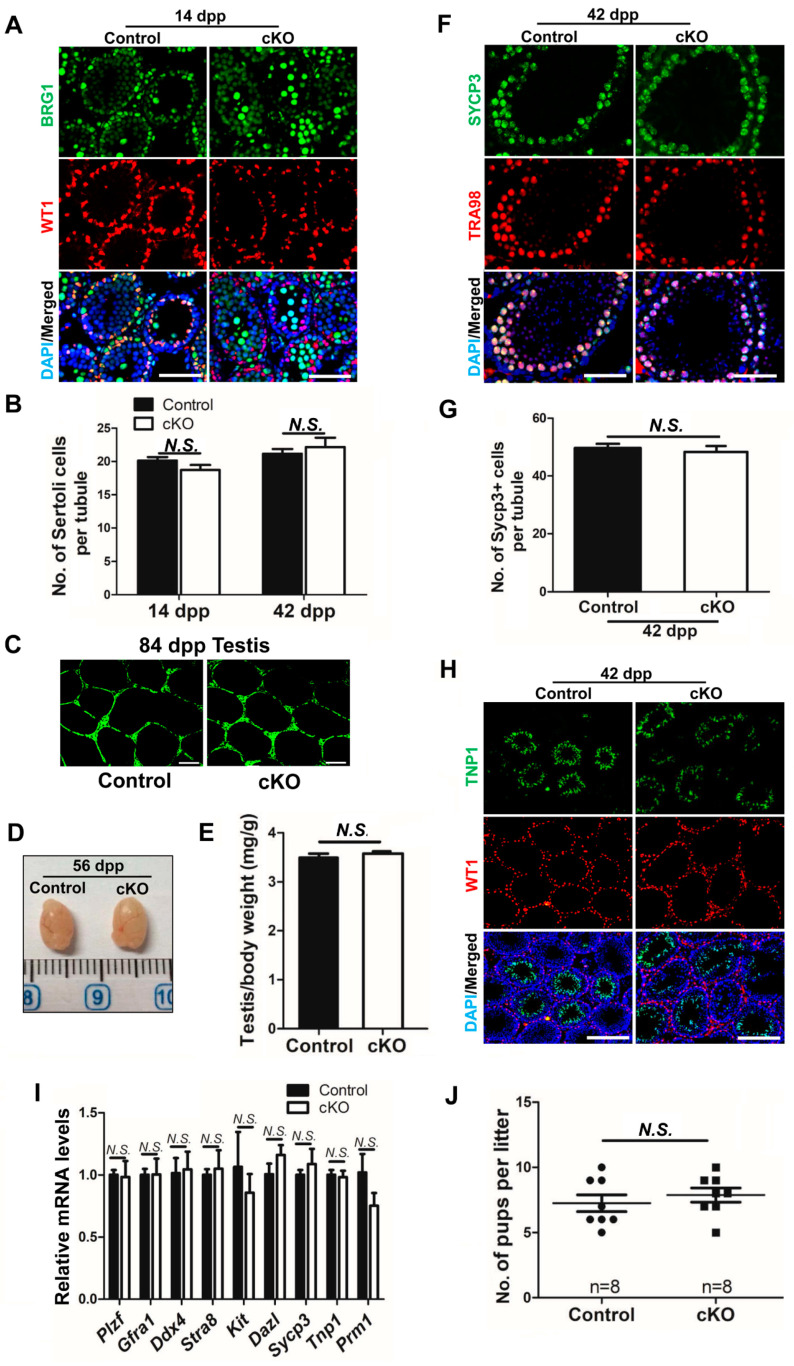
Complete *Brg1* deletion does not affect Sertoli cell development and spermatogenesis. (**A**) Expression of BRG1 and WT1 was examined with IHF in control and cKO testes from mice at 14 dpp. Scale bars, 100 μm. (**B**) Quantification of WT1^+^ Sertoli cells per tubule in the transverse section of the testes from 14 dpp and 42 dpp mice. Data represent mean ± SEM. *n* = 7 for 14 dpp and *n* = 6 for 42 dpp mice. (**C**) Integrity of the BTB was assessed in testes from cKO mice and their littermate controls, as displayed by a biotin tracer (Alexa Fluor 488 fluorescence). Scale bars: 100 μm. (**D**) Representative images of testes from control and cKO mice at 56 dpp. (E) The ratio of testis/body weight was examined in cKO mice and their control littermates at 42 dpp. Data are presented as mean ± SEM. (**F**) Germ cells were examined by IHF, with antibodies against SYCP3 and TRA98, counterstained with DAPI in testes from 42 dpp mice. Scale bars: 100 μm. (**G**) Numbers of SYCP3^+^ spermatocytes per tubule were counted in the transverse section of the testes from 42 dpp wildtype and cKO mice. Data are presented as mean ± SEM, averaged from three transverse sections. (**H**) WT1^+^ Sertoli cells and TNP1^+^ haploid germ cells were examined with IHF in testes from 42 dpp mice. Scale bars, 200 μm. (**I**) Transcript levels of germ cell specific genes were determined by real-time RT-PCR in testis from 56 dpp control and cKO mice. Gene expression was normalized to *Gapdh* and relative expression levels were calculated to *Brg1^f/f^* control group. Data are presented as mean ± SEM from three independent experiments. (**J**) The numbers of pups per litter were averaged from 8 litters of 2 control males and 8 litters from 2 cKO males. n: the number of litters. Data represent mean ± SEM. (**B**,**E**,**G**,**I**,**J**) Statistical analysis was performed using Student’s t-test. *N.S.:* no significance.

To exclude the possibility that existing BRG1^+^ Sertoli cells from partial knockout mice might have masked the requirement of BRG1 in Sertoli cell development, we crossed *Brg1* conditional knockout mice (*Brg1^f/f^*) with another *Amh-Cre* (EMMA) mouse strain originally developed from EMMA, in which CRE expression starts at 15 dpc (see the Reference [29] in Materials and Methods). IHF revealed that the BRG1 protein was completely deleted in all Sertoli cells, but not in the germ cells of *Brg1^f/f^; Amh-Cre* (cKO) mice at 14 dpp (Figure 4A), indicating efficient and specific *Cre* recombinase activities in *Amh-Cre* (EMMA) mice. However, we did not observe obvious changes in the numbers of Sertoli cells upon *Brg1* conditional knockout at 14 dpp and 42 dpp (Figure 4A,B), suggesting that Sertoli cell development is not affected. BTB integrity was further evaluated using the biotin tracer. We found that the green signal was clearly restricted to the interstitial space in both cKO and control testes (Figure 4C). Consistent with these data, the expression of BTB related genes was not altered in *Brg1* deleted Sertoli cells isolated from 14 dpp mice (Figure S3A,B). These observations thus suggest that Sertoli cell development and BTB function are not disrupted upon *Brg1* deletion.

We next evaluated the impact of *Brg1* deletion in Sertoli cells on spermatogenesis and male fertility. No significant difference was observed in the gross morphology of testes between cKO mice and their control littermates at 56 dpp (Figure 4D). The cKO mice had a comparable testes/body weight ratio to their littermate controls at 42 dpp (Figure 4E). We further investigated postnatal germ cell development in these mice with IHF. SYCP3 [30] staining revealed normal spermatocyte development upon *Brg1* conditional deletion in mice at 42 dpp (Figure 4F,G), while TRA98 [31], a germ cell marker, also detected germ cells in testis sections from cKO mice (Figure 4F). In addition, mature germ cells were found in the inner adluminal compartments of seminiferous tubules, similar to those in control mice, as examined by IHF with antibodies against TNP1 [32], which marks haploid spermatids (Figure 4H). Consistent with above findings, transcript levels of germline genes were not altered in *Brg1* deleted testis from 56 dpp mice (Figure 4I). These findings suggest that the spermatogenesis in *Brg1* cKO mice is complete. Finally, we evaluated the impact of *Brg1* deletion in Sertoli cells on male fertility using a breeding testing. We found that the breeding performance of *Brg1* cKO males were comparable to that of control male mice (Figure 4J). In summary, we conclude that *Brg1* is dispensable for Sertoli cell development and their functional contribution to spermatogenesis.

### 2.5. BRM Is Expressed Exclusively in Sertoli Cells

**Figure 5:**
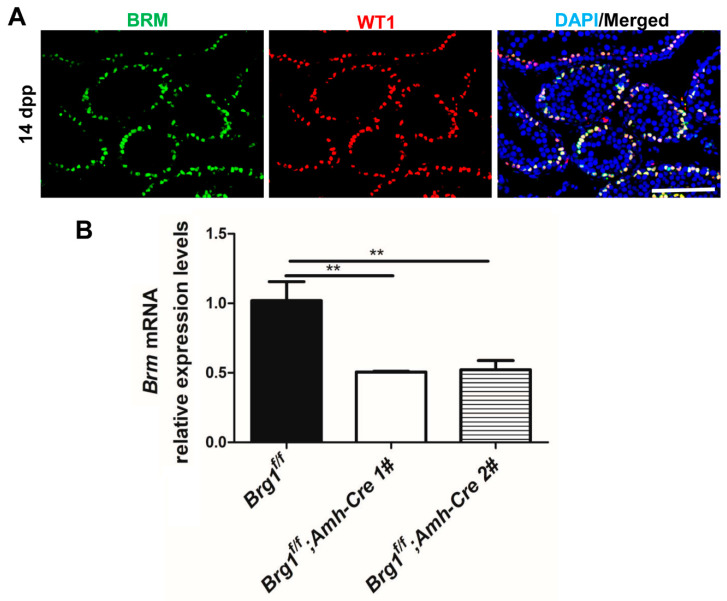
BRM is exclusively expressed in Sertoli cells and *Brm* expression in isolated *Brg1-cKO* Sertoli cells. (**A**) BRM and WT1 expression was examined with IHF in Sertoli cells from 14 dpp mice. Scale bar: 200 μm. (**B**) *Brm* mRNA expression was determined by real-time RT-PCR in isolated Sertoli cells from one control and two *Brg1* cKO (*Brg1^f/f^; Amh-Cre*) mice at 14 dpp. *Brm* expression was normalized to Gapdh and relative expression levels were calculated to *Brg1^f/f^* group. Data are presented as mean ± SEM from two technical replicates. Statistical analysis was performed using Student’s *t*-test. **: *p* < 0.01.

BRG1 and BRM are both essential catalytic unit for SWI/SNF complex. The lack of influence from *Brg1* knockout in Sertoli cells may be the result of the functional compensation from BRM. We thus examined the expression pattern of BRM in testes from mice at 14 dpp. In contrast to BRG1, BRM was expressed solely in somatic Sertoli cells, but not in germ cells (Figure 5A). Interestingly, *Brg1* deletion in Sertoli cells by *Amh-Cre* (EMMA) resulted in a reduction of *Brm* mRNA levels to about 50% (Figure 5B). Future work on Sertoli cell-specific *Brg1* and *Brm* double knockout mice is warranted to determine whether the SWI/SNF complex plays a critical role in regulating Sertoli cell development and functions.

## 3. Discussion

Given the direct physical interaction of Sertoli cells with germ cells in seminiferous tubules, alteration in their number and/or function usually leads to defects in spermatogenesis [4,17,33]. Sertoli cells have a very active transcription and contribute to germ cell development by secreting various signaling molecules and nutrients [4,8,9]. Yet the epigenetic regulation mechanisms underlying Sertoli cell transcription and functions remain to be elucidated. The mammalian SWI/SNF chromatin remodeling complex is critical for tissue development, cell survival, and transcription regulation. To understand the potential roles of SWI/SNF in the epigenetic regulation of Sertoli cell development, we conditionally deleted *Brg1* in Sertoli cells using two different *Amh-Cre* lines. Surprisingly, albeit the critical contribution of BRG1 to embryogenesis, transcriptional regulation, and various biological processes, we observed no obvious changes in number, BTB functioning, and the development of Sertoli cells upon the complete deletion of *Brg1*. These results suggest the functions and contribution of Sertoli cells to germ cell development are not affected by *Brg1* deletion. Indeed, when *Brg1* was deleted in Sertoli cells, we observed complete spermatogenesis and proper male fertility. We thus conclude that BRG1 is dispensable for Sertoli cell development and functions during spermatogenesis.

The mammalian SWI/SNF complex contains either BRG1 ATPase or its homolog BRM as the catalytic subunit. BRG1 and BRM share 86% similarity in their protein sequences, the requirement for BRG1 and BRM in various biological processes, however, is different [34]. In embryonic stem cells (ESCs), ESC-specific SWI/SNF complex is characterized by the presence of BRG1 [35,36,37,38]. *Brg1* deletion impairs ESC self-renewal, which cannot be compensated by BRM [22,35]. By contrast, in neural stem cells (NSCs), the SWI/SNF complex has an alternative catalytic subunit, including either *Brg1* or *Brm* [39,40]. However, despite being expressed at a high level, *Brm* is unable to compensate for loss of function of *Brg1* in NSCs [41]. In addition, BRG1-SWI/SNF complex plays a dominant role during embryonic development [22,26,42]. *Brg1* deletion causes embryonic lethality, while *Brm^–/–^* mice develop normally. Nevertheless, compared to their control littermates, *Brm* mutant mice are ~15% heavier, and their embryonic fibroblasts display defects in cell cycle regulation [26]. In this study, we further examined whether BRM was expressed in Sertoli cells. We found that BRM was exclusively expressed in Sertoli cells, different from the BRG1 protein that was detected in both germ cells and Sertoli cells (Figure 5A). As *Brm* deleted males are fertile [26], we hypothesize that *Brg1* and *Brm* may have redundant roles in mouse Sertoli cell development. Future studies with *Brg1* and *Brm* double knockouts will shed light on the roles of the SWI/SNF chromatin remodeling complex in Sertoli cells during spermatogenesis.

In our study, we used two Sertoli cell specific *Brg1* knockout mouse models, to determine the requirement of *Brg1* for Sertoli cell development and functions. The first model, using an *Amh-Cre* (Jackson) transgenic line, displayed a partial excision of *Brg1* (Figure 2C). Based upon the results from a *Rosa-EYFP* reporter, we confirmed that this *Amh-Cre* (Jackson) line had specific, but inefficient recombinase activity in Sertoli cells. To exclude the possibility that the lack of phenotype in *Brg1* cKO mice was due to partial *Brg1* deletion, we used the second *Amh-Cre* (EMMA) strain which completely removed *Brg1* from Sertoli cells. However, male mice from this mouse model are fertile, thereby suggesting that BRG1 does not play a critical role in the development and functions of Sertoli cells. Although both *Amh-Cre* mice were generated through the pronuclear microinjection of transgene into fertilized eggs, the regulatory elements of these two *Amh-Cre* trangenes are different (see Reference [27,29] in Materials and Methods). In addition, the expression of randomly inserted transgenes at different genomic locations is often subject to chromatin position effects. While the insertion site of the *Amh-Cre* (EMMA) has been identified within the intron 3 of the *Plekha5* gene (http://www.informatics.jax.org/allele/MGI:2450300), the genomic location of the *Amh-Cre* (Jackson) transgene remains unknown. It is thus possible that *Amh-Cre* (Jackson) is not transcriptionally active in a subset of Sertoli cells. Consistent with our findings on those *Amh-Cre* mouse models, Huang et al. also observed the mosaic deletion within the Sertoli cell population when using *Amh-Cre* (Jackson) [43]. However, other studies used the same *Amh-Cre* (Jackson) strain, but did not find such an incomplete *Cre* recombinase activity [44,45,46]. One potential explanation for this inconsistent observation may relate to different genomic loci of various floxed alleles. The chromatin state of some floxed genomic loci may prevent CRE from accessing the loxP sites. Alternatively, a genetic drift of the *Cre* transgene may occur over time. As a mouse line is continuously intra-bred within the colony, mutations may arise and leads to reduced CRE expression. Therefore, properly maintained and characterized mouse models are crucial to permit successful gene manipulation in reproductive research.

## 4. Materials and Methods

### 4.1. Mouse Lines, Animal Care and Fertility Test

Mice harboring the floxed *Brg1* allele are kind gifts from Drs. Ajeet Singh and Trevor Archer at National Institute of Environmental Health Sciences, National Institute of Health [47]. Two *Amh-Cre* mouse lines were used. One is a generous gift from Dr. Qinghua Shi from University of Science and Technology of China (originally developed from Jackson Laboratory, Bar Harbor, ME, USA, Stock ID: 007915), and another was purchased from Shanghai Model Organisms Center Inc (originally developed from EMMA mouse repository; EMMA mouse repository ID: EM:00022) [27,29]. *Rosa26-EYFP* reporter mouse is a kind gift from Dr. Yan Zhang at the Institut Pasteur of Shanghai, Chinese Academy of Science. All these mouse lines have been described previously [28]. To generate the Sertoli cell specific deletion of *Brg1*, male conditional *Brg1* knockout mice (*Brg1^f/f^*) were bred with female *Brg1^f/f^; Amh-Cre*, and the progeny mice were genotyped with PCR analyses. For the fertility test, adult males were housed with wild-type C57BL/6 females for at least 2 months. The size of litters generated by these males was recorded. All animal experimental procedures (Protocol ID: AR2013/05007) were conducted in accordance with the local Animal Welfare Act and Public Health Service Policy and approved by the Committee of Animal Experimental Ethics at East China Normal University.

### 4.2. Genotyping

Genomic DNA was isolated according to a published protocol [48]. Briefly, tail tips were digested at 95 °C in buffer A (25 mM NaOH, 0.2 mM EDTA, pH 5.0) for 30 min, and the reactions were stopped by adding buffer B (40 mM Tris, pH 5.0). The supernatant was collected for downstream PCR genotyping with Taq Master Mix (Vazyme Biotech, Nanjing, China, P112-AA), according to the manufacturer’s instructions. Sequences of primers and size of products are provided in Table S1.

### 4.3. Histology and Immunohistofluorescence (IHF)

Testes were fixed in Bouin’s solution (Sigma-Aldrich, St. Louis, MO, USA, HT10132) for histology study with hematoxylin (Sigma-Aldrich, MHS16) and eosin (Sigma-Aldrich, HT110216) staining, or fixed in 4% paraformaldehyde (PFA) overnight for IHF. Fixed testes were dehydrated, treated with xylene, embedded in paraffin, and processed into 5 µm sections for hematoxylin and eosin staining. For IHF, testis sections went through antigen retrieval by boiling in 10 mM sodium citrate, pH 6.0, for 20 min, followed with incubation in 10% Fetal Bovine Serum (FBS)/0.5% Tween-20/PBS for 1 hr at room temperature. Samples were further blotted with primary antibodies in 0.1% Tween-20/PBS (PBST) overnight at 4 °C, washed with 0.1% PBST, incubated with Alexa Fluo 488 or 594-conjugated secondary antibodies in 1% BSA/PBS, and counterstained with DAPI/ProLong™ Diamond Antifade Mountant (Thermo Fisher Scientific, Eugene, OR, USA, P36966). All images were collected using a Leica DM4000-B-LED microscope, and processed with Image, J. Antibodies in this study: BRG1 (ab110641), WT1 (ab212951), GFP (ab183734), TRA98 (ab82527), SYCP3 (ab15093), SYCP3 (ab97672), TNP1 (ab73135), and BRM (ab15597) were purchased from Abcam (Cambridge, MA, USA). PLZF (sc-28319) was purchased from Santa Cruz Biotechnology (Dallas, Texas, USA). Alexa Fluor^®^ conjugated secondary antibodies came from Jackson ImmunoResearch Laboratories.

### 4.4. Sertoli Cell Isolation

Sertoli cells were isolated following a published protocol with a minor modification [49]. Briefly, testes from mice at 14 dpp were manually dissected into small clumps and incubated with 1 mg/mL Collagenase IV/2 μg/mL DNase I/DMEM/F12 solution at room temperature for 5 min, with gently pipetting up and down several times. Fragmented tubules were precipitated by gravity and digested in 0.25% Trypsin/200 μg/mL DNase I solution at room temperature, with occasional pipetting. The dissociated cells are collected by centrifugation at 300× *g* for 7 min, washed with PBS, and incubated in 10%FBS/DMEM/F-12 at 37 °C with 5% CO_2_. Unattached germ cells in the medium and adherent Sertoli cells were collected separately after 24 h for further analyses in Figure S3A. Finally, purified Sertoli Cells which attached to the culture dishes were obtained.

### 4.5. Total RNA Extraction, Reverse Transcription, and Real-Time PCR

Total RNA was isolated from cells using a RNAiso Plus solution (TAKARA, Dalian, China, 9109), and reverse transcribed into cDNA using a PrimeScrip RT reagent Kit (TAKARA, RR037A). A real-time PCR was performed with FastStart Universal SYBR Green Master (Roche Life Science, Mannheim, Germany, 04913914001) and target gene specific primers, using a Thermo Scientific QuantStudio PCR machine. The data was analyzed using the comparative threshold cycle (ΔΔ*C*t) method and normalized to *Gapdh*. All PCR primers used are listed in Table S2.

### 4.6. Blood-Testis Barrier Integrity Assay

The biotin tracer assay was performed to test the BTB permeability as previously described, with minor modifications [50]. Briefly, anesthetized mice were injected with 50 uL of freshly prepared 10 mg/mL EZ-Link Sulfo-NHS-LC-Biotin (Thermo Scientific, 21335) in 1 mM CaCl_2_/PBS into interstitial space of testes. Animals were euthanized after 60 min. Testes were immediately removed and embedded in OCT (SAKURA, Torrance, CA, USA, 4583). Then, 6 μm-thick cryosections of testes were incubated with Alexa Fluor 488-conjugated streptavidin (Thermo Scientific, 21832) for 30 min at room temperature. Fluorescence images of the cryosections were captured using a Leica DM4000-B-LED microscope. All images were processed with ImageJ.

### 4.7. Statistics

All statistical analyses to determine significance of group differences in this study were performed using Student’s t-test, calculated with GraphPadPrism5 software (GraphPad Software, La Jolla, CA, USA). Results are presented as mean ± SEM. The statistical significance was defined as *: *p* < 0.05; **: *p* < 0.01; ***: *p* < 0.001.

## Supplementary Materials

Supplementary materials can be found at https://www.mdpi.com/1422-0067/21/12/4358/s1.
