# Supplementary material for: BRG1 Is Dispensable for Sertoli Cell Development and Functions in Mice
Source: Int J Mol Sci. 2020 Jun 19;21(12):4358. doi: 10.3390/ijms21124358 (PMC7353015; doi:10.3390/ijms21124358)
Supplement: Supplementary file 1 [file ijms-21-04358-s001.pdf]

BRG1 is dispensable for Sertoli cell development in mice

Shuai Wang[1], Pengxiang Wang[1], Dongli Liang[1*] and Yuan Wang[2*]

[1] Shanghai Key Laboratory of Regulatory Biology, Institute of Biomedical Sciences and School of Life Sciences, East China Normal University, Shanghai, 200241, China

[2] Department of Animal Sciences, College of Agriculture and Natural Resources, Michigan State University, East Lansing, MI 48824, USA

[*] To whom correspondence should be addressed:

Yuan Wang, Department of Animal Sciences, College of Agriculture and Natural Resources, Michigan State University, East Lansing, USA, 48824
Email: wangyu81@msu.edu

Dongli Liang, School of Life Sciences, East China Normal University, Shanghai, China, 200241
Email: dlliang@bio.ecnu.edu.cn

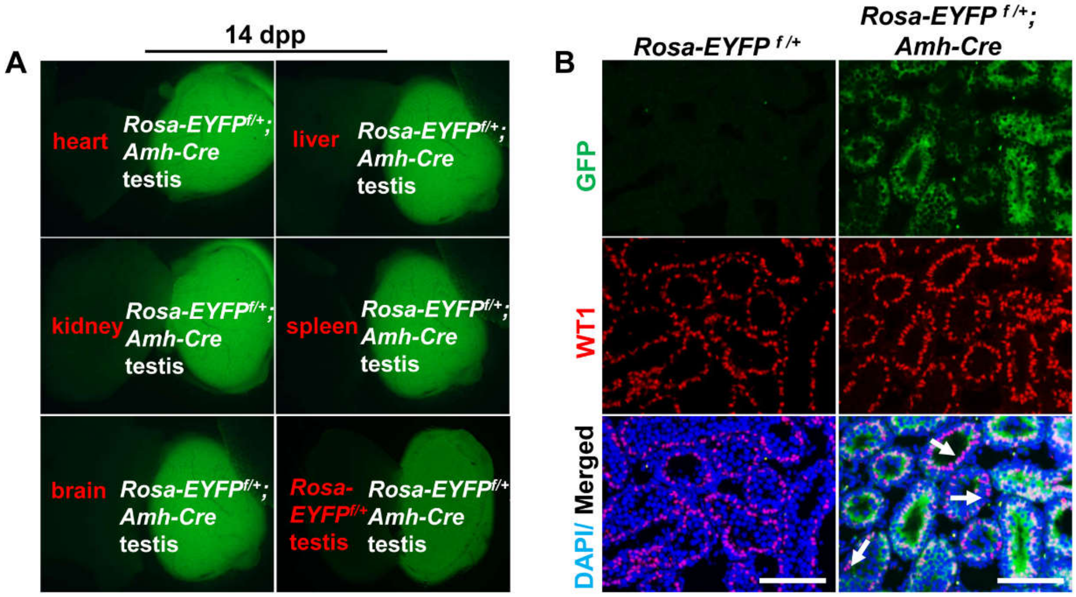

Figure S1. Specific but inefficient CRE recombinase activity in Amh-Cre mice from Jackson Lab.

(A) GFP fluorescence in different organs from *Rosa-EYFP*$^{f/+}$*; Amh-Cre* and *Rosa-EYFP*$^{f/+}$mice.

(B) GFP and WT1 expression was examined with IHF in testes from *Rosa-EYFP*$^{f/+}$*; Amh-Cre* and *Rosa-EYFP*$^{f/+}$mice. CRE in Sertoli cells excised stop codon before EYFP coding sequences, which led to expression of EYFP (detected by a GFP antibody because of high homologous sequences between GFP and EYFP proteins). GFP protein was not expressed in a fraction of Sertoli cells (white arrows), suggesting that *Amh-Cre* recombinase activity was inefficient. Scale bars, 200 µm.

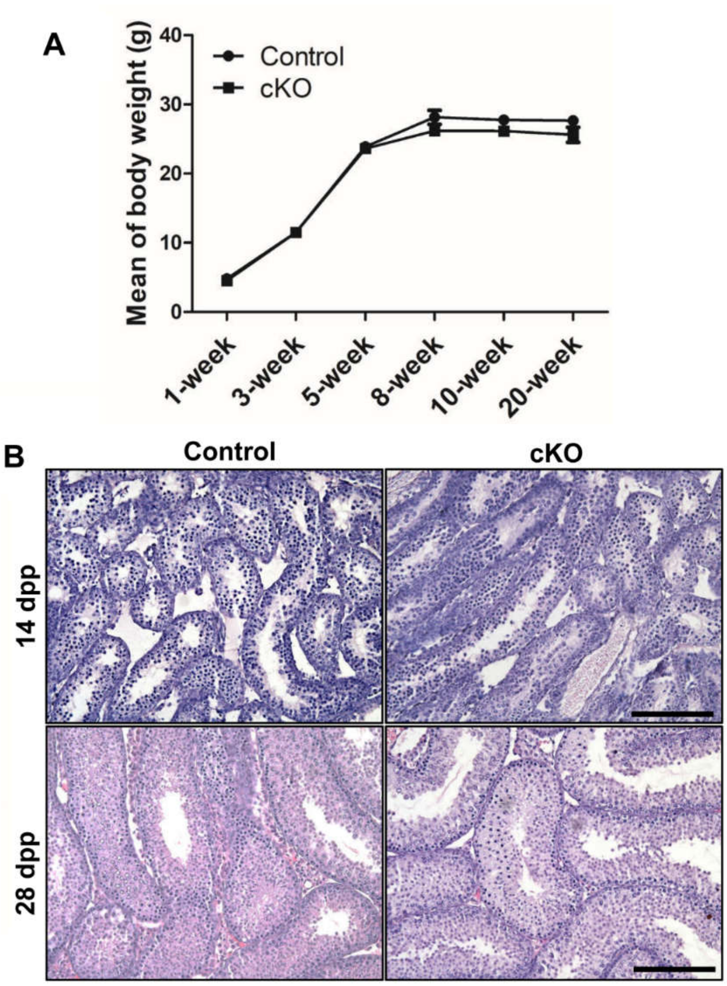

Figure S2. Normal spermatogenesis and body weights in cKO mouse.
(A) Mean body weight of mice at different ages, body weights were recorded for 3–10 animals per group.
(B) Histology study on testes from control and *Brg1*-cKO mice at 14 dpp and 28 dpp. Normal spermatogenesis and germ cell layout in seminiferous tubules were observed in control and cKO testes. Scale bars, 200 µm.

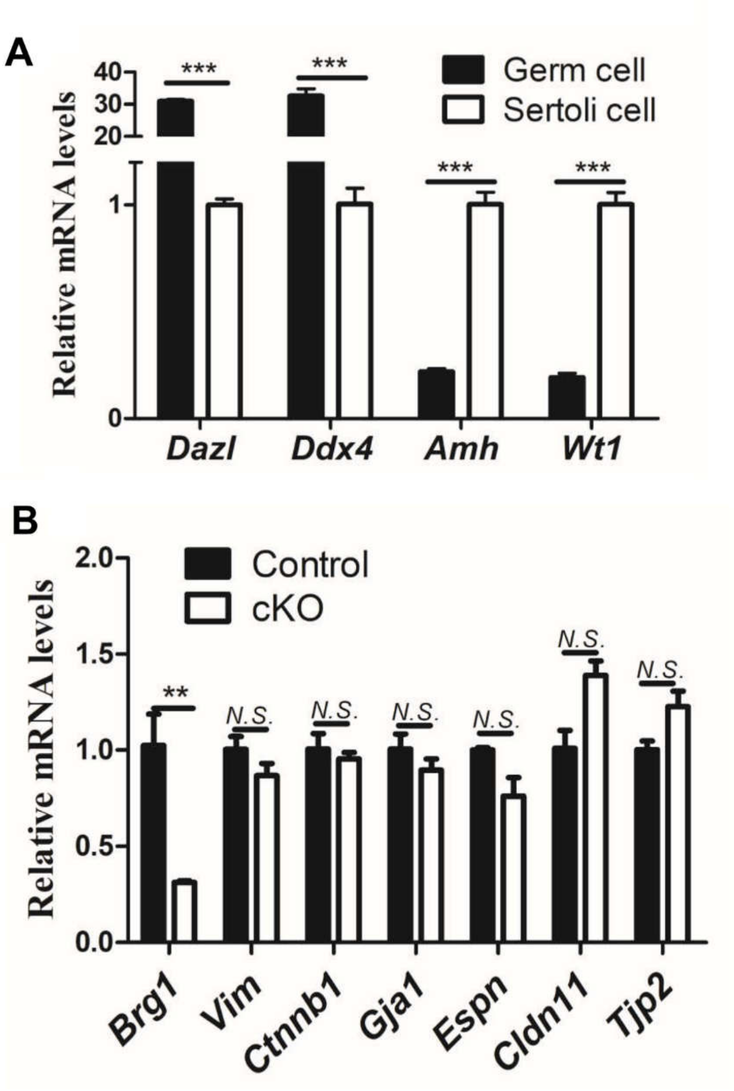

Figure S3. High purity of the isolated Sertoli cells and BTB related-gene expression analysis.

(A) Expression levels of germ cell markers (*Dazl* and *Ddx4*) and Sertoli cell genes were examined by real-time RT-PCR in isolated germ cells and Sertoli cells.

(B) *Brg1* and BTB related-genes were determined for their transcript levels by real-time RT-PCR in isolated Sertoli cells from 14 dpp control and cKO mice. (A-B) Data are presented as mean ± SEM from three independent experiments. **: $p < 0.01$; ***: $p < 0.001$; *N.S.*: no significance.

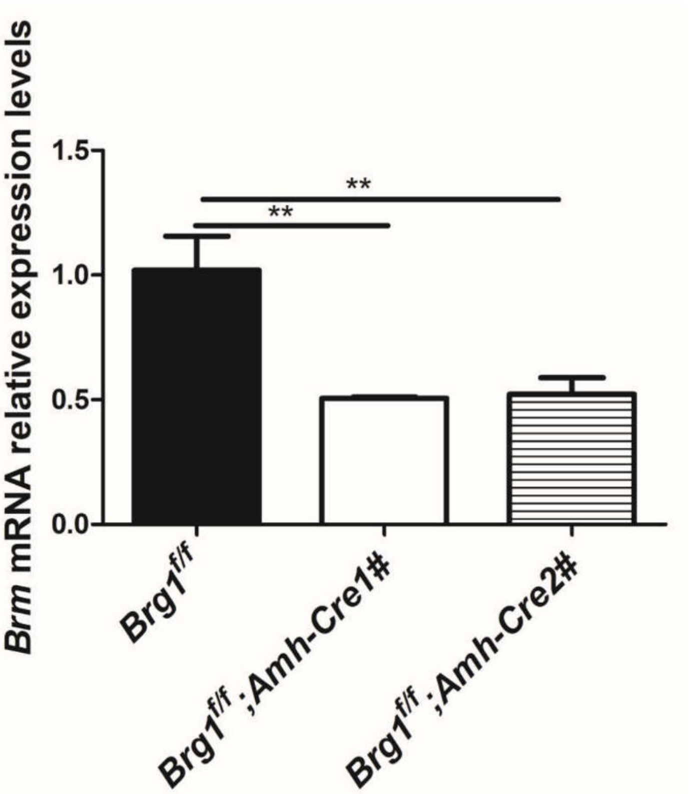

Figure S4. Brm expression in isolated Brg1-cKO and control Sertoli cells. *Brm* mRNA expression was determined by real-time RT-PCR in isolated Sertoli cells from control and cKO mice at 14 dpp. *Brm* expression was normalized to *Gapdh* and relative expression levels were calculated to *Brg1*$^{f/f}$ group. Data are presented as mean ± SEM. **: $p < 0.01$.

Table S1. Primers used in genotyping

Gene name	Forward primers	Reverse primers	Amplicon Size
Brg1	GTCATACTTATGTCATAGCC	GCCTTGTCTCAAACTGATAAG	Homozygous:380bp Wildtype:250bp
Amh (Jackson)	GCGGTCTGGCAGTAAAAACTATC	GTGAAACAGCATTGCTGTCACTT	100bp
Amh (EMMA)	TCCTGGAAAATGCTTCTGTCCG	CAGGGTGTTATAAGCAATCCC	400bp

Table S2. Primers used in real-time PCR

Gene name	Forward primers	Reverse primers
Brg1	GGTTCTGCCCACAGCATGAT	GGACTCCATAGGCTTGTGCAT
Brm	CTCCTGGACCAATTCTGGGG	CATCGTTGACAGAGGATGTGAG
Dazl	ATGTCTGCCACAACTTCTGAG	CTGATTTCGGTTTCATCCATCCT
Ddx4	GCTTCATCAGATATTGGCGAGT	GCTTGGAAAACCCTCTGCTT
Amh	CCACACCTCTCTCCACTGGTA	GGCACAAAGGTTCAGGGGG
Wt1	GAGAGCCAGCCTACCATCC	GGGTCCTCGTGTTTGAAGGAA
Vim	CGGCTGCGAGAGAAATTGC	CCACTTTCCGTTCAAGGTCAAG
Ctnnb1	ATGGAGCCGGACAGAAAAGC	CTTGCCACTCAGGGAAGGA
Gja1	ACAGCGGTTGAGTCAGCTTG	GAGAGATGGGGAAGGACTTGT
Espn	CCACAGGCTACCTCTCTTGC	AGCAGCCACTTCACCACATC
Cldn11	ATGGTAGCCACTTGCCTTCAG	AGTTCGTCCATTTTTCGGCAG
Tjp2	ATGGGAGCAGTACACCGTGA	TGACCACCCTGTCATTTTCTTG
Gapdh	AGGTCGGTGTGAACGGATTTG	TGTAGACCATGTAGTTGAGGTCA
